# Restoring functional neurofibromin by protein transduction

**DOI:** 10.1038/s41598-018-24310-5

**Published:** 2018-04-18

**Authors:** K. Mellert, S. Lechner, M. Lüdeke, M. Lamla, P. Möller, R. Kemkemer, K. Scheffzek, D. Kaufmann

**Affiliations:** 1 Present Address: Institute of Pathology, University of Ulm, Ulm, Germany; 2 Division of Biological Chemistry, Biocenter, Innsbruck Medical University, Innsbruck, Austria; 3 Institute of Human Genetics, University of Ulm, Ulm, Germany; 4 Institute of Organic Chemistry III, Ulm University, Ulm, Germany; 5 Department of New Materials and Biosystems, Max Planck Institute for Medical Research, Stuttgart, Germany; 6 Present Address: Reutlingen University, Applied Chemistry, Reutlingen, Germany

## Abstract

In Neurofibromatosis 1 (NF1) germ line loss of function mutations result in reduction of cellular neurofibromin content (*NF1*+/−, *NF1* haploinsufficiency). The Ras-GAP neurofibromin is a very large cytoplasmic protein (2818 AA, 319 kDa) involved in the RAS-MAPK pathway. Aside from regulation of proliferation, it is involved in mechanosensoric of cells. We investigated neurofibromin replacement in cultured human fibroblasts showing reduced amount of neurofibromin. Full length neurofibromin was produced recombinantly in insect cells and purified. Protein transduction into cultured fibroblasts was performed employing cell penetrating peptides along with photochemical internalization. This combination of transduction strategies ensures the intracellular uptake and the translocation to the cytoplasm of neurofibromin. The transduced neurofibromin is functional, indicated by functional rescue of reduced mechanosensoric blindness and reduced RasGAP activity in cultured fibroblasts of NF1 patients or normal fibroblasts treated by NF1 siRNA. Our study shows that recombinant neurofibromin is able to revert cellular effects of *NF1* haploinsuffiency *in vitro*, indicating a use of protein transduction into cells as a potential treatment strategy for the monogenic disease NF1.

## Introduction

In monogenic dominantly inherited diseases such as Neurofibromatosis type 1 (NF1), a loss of function mutation in one *NF1* allele leads to a reduced amount of the functional corresponding protein in all body cells expressing this gene. The *NF1* gene product, the protein neurofibromin, is involved in the regulation of development and growth of a variety of tissues^1^. Several NF1 tumour manifestations are due to the entire cellular loss of functional neurofibromin by a stochastic inactivating mutation in the (second) *NF1* wild type allele^2^. Aside from *NF1* loss in specific cell populations (e.g. Schwann cells) an *NF1* heterozygous microenvironment is critical for the formation of NF1 tumours like plexiform neurofibromas or optic gliomas^3–5^. *NF1*-haploinsufficiency itself also becomes manifest in several non-tumour symptoms in NF1 patients as the cognitive impairment, the general hyperpigmentation, bone disorders or the NF1 vasculopathy^5^. Cellular effects of neurofibromin reduction were also demonstrated *in vitro* in several cell types such as fibroblasts, melanocytes, keratinocytes, osteoblasts, astrocytes, and hematopoietic cells^5–8^. *NF1*+/− fibroblasts have a reduced capability of orientating themselves on nano-microstrucured surfaces indicating a function of neurofibromin in mechanosensoric regulation of such cells^9^. There is no systemic therapy for the whole clinical disease pattern of NF1. Treatments are yet based on symptomatic surgical interventions of the NF1 tumours and on improving the NF1 specific learning disabilities by drugs^10,11^. A phase 1 study of children with NF1 treated with the MEK inhibitor Selumetinib revealed promising reduction of the plexiform neurofibrosarcoma tumour mass in 17 of 24 patients^12^. Until now, there is no successful genetic therapy of NF1. Restoring functional neurofibromin in protein transduction approaches in all patient body cells as an alternative might be a clue to avoid the risks of genetic gene replacement therapies^13^.

In autosomal recessive transmitted diseases protein replacement therapies have been tested in patients with Morbus Gaucher^14^, Fabry’s disease^15,16^, Mucopolysaccharosidosises, Morbus Pompe or Hemophilia A^17–19^. These diseases are caused by an entire lack of a protein with extracellular effects or a function in lysosomes in cells.

Neurofibromin is a 320 kDa cytoplasmic protein^20,21^ containing a central RasGAP related domain^22–24^ followed by a bipartite glycerophospholipid binding module composed of an N-terminal Sec. 14- and a C-terminal pleckstrin homology (PH) like domain^25–27^. The use of protein replacement in therapies related to a protein with an intracellular cytosolic function is limited by the lysosomal disintegration of the proteins that were taken up into cells as demonstrated in introducing functional antibodies in cultured cells^28^. A technique to induce cytoplasmic uptake of proteins *in vitro* is the protein transduction using specialized protein transduction reagents. This technique is widely used to transport toxic agents into tumour cells^29,30^. However, we could show that protein transduction using different commercial protein transduction reagents primarily leads to an endosomal cellular uptake^31^. Cotreating cells with a photosensitizer and light induced disruption of endosomes and lysosomes called photochemical internalization (PCI) leads to a cytosolic distribution of the internalized proteins *in vitro*.

Here, we applied this transduction techniques to test neurofibromin incorporation in cultured *NF1* haploinsufficient cells, either *NF1*+/− fibroblasts derived from NF1 patients showing a reduced amount of neurofibromin^32^ or human fibroblast derived from a healthy donor treated with *NF1* siRNA. Full length neurofibromin (synNF1) was expressed in insect cells and purified and characterized following Dunzendorfer-Matt *et al*.^33^. We investigated whether synNF1 replacement could revert cellular effects of *NF1* haploinsuffiency *in vitro*. One effect of *NF1* haploinsuffiency used as first read out is the relative mechanosensoric blindness to micro-nano-structures measured as reduced capability to orientate themselves on polydimethysiloxane (PDMS) gels structured with parallel elevations of 200 nm height and a width of 2 µm, as described for fibroblasts derived from *NF1* patients^9^. We tested if this effect could be provoked in normal fibroblasts by reducing the *NF1* message with *NF1* siRNAs and rescuing the induced effects by neurofibromin transduction. The second read out for *NF1* haploinsufficiency is the altered RasGAP activity measured by the amount of phosphorylated ERK1/2, a downstream effector in the Ras-Raf-MAPK pathway regulated by the neurofibromin RAS-GAP activity^34,35^. Our investigations show that treatment with recombinant neurofibromin reverts these cellular effects of *NF1* haploinsuffiency *in vitro*.

## Results

### Timescale of experimental setup

We have previously described a reduced orientation capability of *NF1*+/− fibroblasts cultured on PDMS nano-micro structures of parallel groves at a height of 200 nm and a width and interspace of 2 µm^9^. To develop an optimal experimental time scale for the following transduction experiments fibroblasts of 3 healthy donors (*NF1*+/+) and 3 NF1 patients (*NF1*+/−) were seeded on the structured gels for 24, 48 and 96 hours. The highest difference between *NF1*+/+ and *NF1*+/− was detected when the cells could orientate themselves for 48 hours (Table 1).

**Table 1:** Determination of the optimal time of fibroblasts orientating themselves to nano-micro structures.

Experiment	24 hrs	48 hrs	96 hrs
A. NF1+/+	1,06	0,97	0,96
NF1+/−	1,13	1,55	1,54
B. NF1+/+	0,94	1,03	1,04
NF1+/−	1,01	1,37	0,78
C. NF1+/+	1,00	1,00	n.d.
NF1+/−	1,75	2,25	n.d.
NF1+/+	1,00 ± 0,06	1,00 ± 0,03	1,00 ± 0,05
NF1+/−	1,30 ± 0,40	1,72 ± 0,46	1,16 ± 0,54
p-value	**0**,**14**	**0**,**03**	**0**,**36**

The normalized orientation angle 24, 48 and 96 hours after seeding the fibroblasts on micro-nano-structures is given. Each experiment (A, B, C) consisted of >100 single cells each. Experiments were parallelized by seeding *NF1*+/+ and *NF1*+/− fibroblasts of the comparable passage numbers in equal numbers at the same time. Significance of the differences was determined using two-sided t-tests.

**Figure 1:**
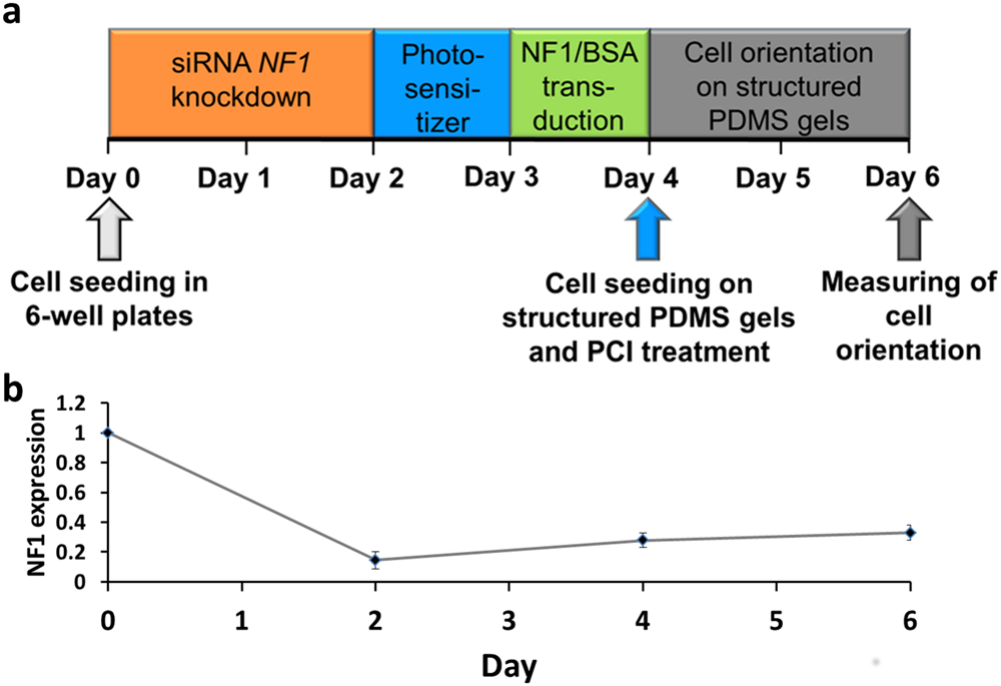
Time scale of transduction experiments. (**a**) Time scale of experimental combination of siRNA based *NF1* knockdown, protein transduction, photochemical internalization and cell orientation of nano-micro structured surfaces. The fibroblasts were seeded in 6 well plates and a siRNA based *NF1* knockdown was performed for 2 days (Day 0 to Day 2). Then, the fibroblasts were incubated with the photosensitizer TPPS_4_ for 24 hours (Day 2 to Day 3) followed by a protein transduction of another 24 hours (Day 3 to Day 4). The blue arrow represents a 10 minute illumination time to perform the photochemical internalization treatment while the cells were seeded onto nano-micro structured PDMS gels. After an orientation time of 48 h (Day 4 to Day 6), the fibroblasts were fixed and stained with DAPI and the orientation of the cells hours was measured. (**b**) The levels of *NF1* mRNA were measured in *NF1*+/+ fibroblasts (KF3) transfected with control siRNAs (Day 0), after 48 hours of *NF1* siRNA (siNF1) transfection (Day 2) and after 48 and 96 hours of recovery posterior to the siRNA transfection (Day 4 and Day 6 respectively). After the initial treatment, the KF3 fibroblasts showed a significant decrease in *NF1* mRNA levels (n = 3). Error bars indicate +/− 1 SD.

Experiments concerning protein transduction of the fluorescently labeled model drug Atto488-BSA showed that good results could be achieved when the transduction time was 18–24 hours. The combined treatment with photochemical internalization (PCI) to release endosomally trapped protein to the cytosol of the cells required 24 hours preincubation time with the photosensitizer TPPS_4_^31^. Therefore, experiments combining protein transduction, PCI and cellular orientation on nano-micro structured surfaces were designed to last 96 hours as shown in the experimental timescale **(**Fig. 1a**)**. In case of a siRNA *NF1* knockdown, additional 48 hours were prepended.

### siRNA based *NF1* knockdown induces the partial blindness to micro-nano-structured PDMS gels in cultured fibroblasts

The partial blindness to micro-nano-structured PDMS gels was characterized in primary *NF1*+/− fibroblasts derived from NF1 patients^9^. To ensure that this cellular phenotype is related to the expression of *NF1*, siRNA-based *NF1* knockdown experiments were performed in fibroblasts of a healthy human donor (*NF1*+/+; KF3). The neurofibromin half-life period is about 25 hours^32,36^. Therefore, the *NF1*+/+ fibroblasts were transfected for 48 hours either with *NF1* specific siRNAs or with control siRNA (Qiagen, Hilden; Supplementary Table 1) using the Lipofectamine RNAiMax transfection reagent (Invitrogen). The amount of *NF1* transcripts was measured by RT qPCR after 48, 96 and 144 hours. After the initial treatment, the KF3 fibroblasts showed a decrease in *NF1* mRNA levels to 15%, followed by a slow recovery to 33% **(**Fig. 1b). The transfection with control siRNA (Qiagen, Hilden) itself did not change the *NF1* message significantly (Supplementary Figure 1).

**Figure 2:**
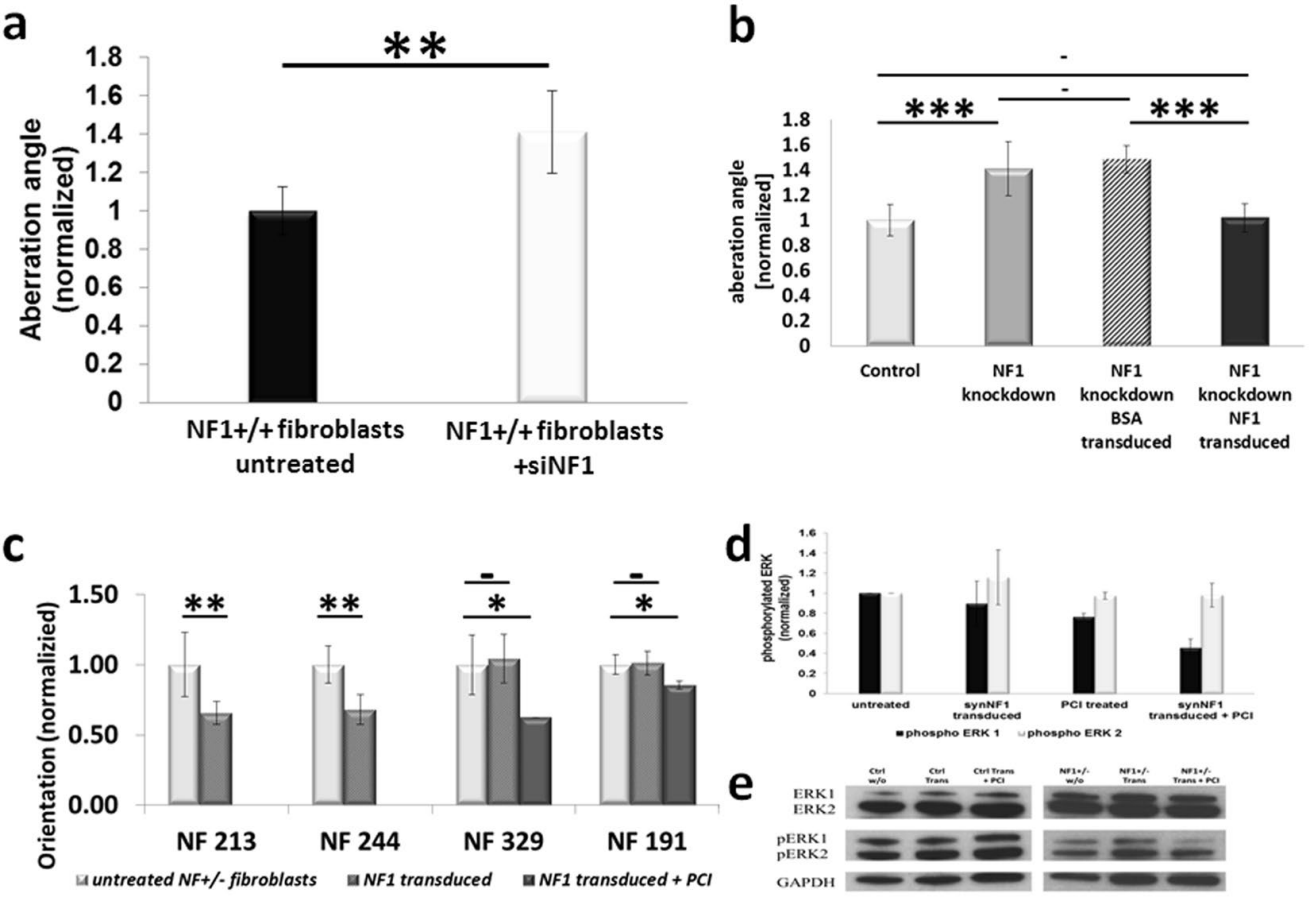
Cellular orientation experiments and measurement of the impact of synNF1 on the phosphorylated ERK ½ levels. (**a**) Orientation of fibroblasts with NF1 knockdown. SiRNA based NF1 knockdown results in a decreased orientation capability of control fibroblasts. The orientation to nano-micro structured PDMS surfaces of KF3 fibroblasts was determined by measuring the median aberration angles of the cells either without any treatment (KF3 untreated) or posterior to a siRNA based NF1 knockdown (KF3 +siNF1). Given are the means +/− 1 SD (n = 5, 100–700 single cells each). (**b**) Functional rescue of the reduced orientation capability of siNF1 transfected KF3 fibroblasts by Neurofibromin (synNF1) transduction. The aberrations angles of KF3 fibroblasts to nano-micro structures of PDMS gels were measured and normalized to the mean of the measured untreated cells (KF3 untreated). Shown are the means of 3 to 5 independent experiments consisting of 100 to 700 individual cells each. Error bars represent the standard deviation of the independent experiments. (**c**) Improvement of the orientation capability in NF1 patient fibroblasts by synNF1 transduction. The measured median aberration angle to given structured surfaces was measured without protein transduction (untreated), after transduction of synNF1 using Endo-Porter (synNF1 transduced) and for the non-responder after a combination treatment of synNF1 transduction follow by a photochemical internalization treatment (synNF1 treated +PCI). Error bars represent the standard deviation of the means of 3–5 independent experiments (each consisting of 100–700 single cells). (**d**) Transduction of synNF1 into NF1 patient fibroblasts results in a decrease of the phosphorylation of ERK1 but not of ERK2. Normalized amounts of phosphorylated ERK1/2 in NF1 patient fibroblasts without treatment, the single treatments (synNF1 transduced, PCI treated) or the combined treatments. The bars represent the means of the measured phosphoErk levels in fibroblasts of the 2 different patients NF191 and NF244 (3 independent experiments each). Error bars indicate +/− 1 standard deviation. (**e**) Example of a western blot measurement of levels of pERK with NF+/+ (crtl) and NF1+/− cells (NF1+/−) either untreated (w/o), synNF1 transduced (Trans) or cotreated with synNF1 transduction and photochemical internalisation (Trans + PCI). Stars indicate the level of significance (*p < 0.05; **p < 0.01; ***p < 0.001) two-sided T-test).

The long-term *NF1* mRNA silencing allows the experimental setup for combined protein transduction, PCI and measurement of the orientation on structured surfaces. The *NF1* siRNA treated *NF1*+/+ fibroblasts (KF3) showed a reduced capability in orientating on the tested micro-nano-structured surfaces **(**Fig. 2a**)**. The median deviation angle of the fibroblasts rose to about 1.4 fold (+/−0.21; p < 0.01) compared to fibroblasts transfected with control siRNA. These findings fit to experiments with primary *NF1*+/− and age matched NF1+/+ fibroblasts^9^. These data demonstrate that the *NF1* siRNA silencing in fibroblasts results in the cellular phenotype of partial mechanosensoric blindness.

### Production of synNF1 and syn NF1EGFP

SynNF1 was expressed and purified as described^33^. The purified protein is biochemically active and elutes as a putative dimer in size exclusion chromatography. Based on synNF1, a 3xeGFP fused neurofibromin version was constructed with 3 eGFPs in tandem connected to the C-terminal end, expressed and purified as described in Methods.

### SynNF1-eGFP can be transduced into fibroblasts using different transduction reagents

**Figure 3:**
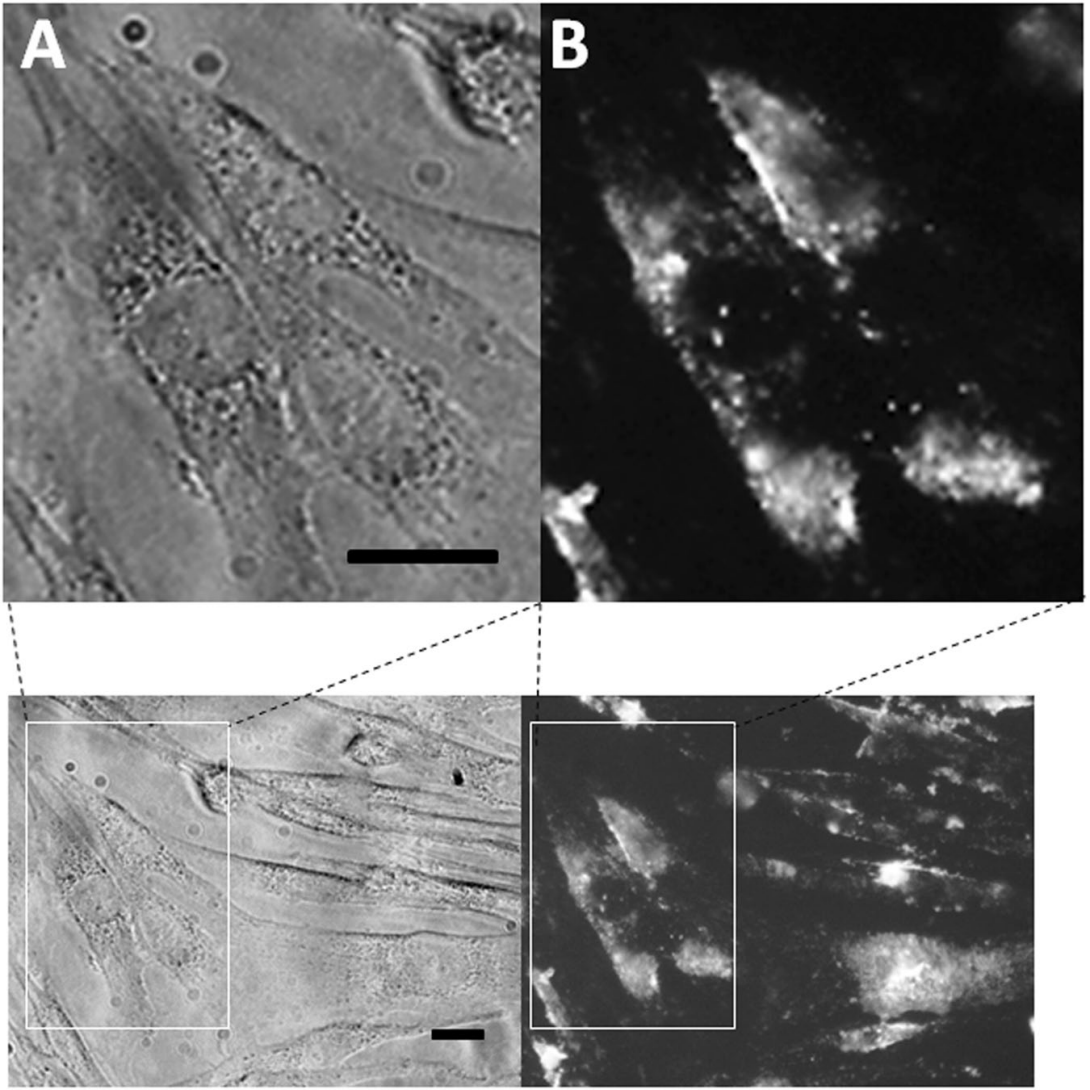
Detection of transduced synNF1eGFP in KF3 fibroblasts. (**A**) Phase contrast picture of fibroblasts transduced with synNF1eGFP using the Endo-Porter transduction reagent. (**B**) Fluorescence image of the same cells using a fluorescence filter set for green fluorescent protein. The bar indicates a length of 10 µm.

Recent transduction experiments using fluorescently labelled BSA comparing different transduction reagents revealed that the transduction efficiency was optimal using cell penetrating peptides^31^. Therefore, we used the cell penetrating peptide Chariot (Active Motif, Carlsbad, CA, USA) to transduce a recombinant and 3xeGFP fused neurofibromin (synNF1-eGFP) protein into fibroblasts of a healthy control person (KF3). Live cell imaging showed that the synNF1eGFP was transduced efficiently into the fibroblasts and, in accordance to the comparative study, could be detected in punctate structures indicating a predominantly endosomal uptake of the transduced protein. However, the fluorescent signals were relatively weak (data not shown). Using the endocytosis interfering transduction reagent Endo-Porter (Gene Tools, LLC) led to stronger fluorescence signals within the fibroblasts after transduction of synNF1-eGFP both in fixed and living cells **(**Fig. 3**)**.Therefore, in the following experiments, Endo-Porter was used as transduction reagent. Because several of the signals were punctuated, an additional photochemical internalization (PCI) after transduction was added to the synNF1-eGFP transduction. This treatment leads to a release of endolysosomal entrapped proteins^37^.

### SynNF1 rescues the orientation capability fibroblasts with *NF1* knockdown

KF3 fibroblasts show a decrease in their orientation after siRNA mediated NF1 knockdown in combination with a transduction with Atto488-BSA as a control protein and a PCI treatment in comparison to the untreated cells **(**Fig. 2b**)**. Replacing the model compound BSA by synNF1 leads to a full rescue of the orientation competence of the cells. Though, the transduced synNF1 is able to resume the functions of neurofibromin in fibroblasts with a *NF1* knockdown.

### Partial blindness to submicron structures in patient fibroblasts is decreased by synNF1 transduction

In fibroblasts derived from 5 NF1 patients we tested if the transduction of synNF1 can improve the cells’ ability to orientate themselves on given topographies. In 3 of 5 patients (NF213, NF244 and NF282) the synNF1 transduction using the Endo-Porter transduction reagent (Gene Tools) exclusively led to a distinct diminishment of the median aberration angle between the cells and the surface structures and therefore to a better orientation of the cells. In the 2 of 5 patient fibroblasts (NF329 and NF191) that did not respond to the synNF1 transduction an improvement of the orientation capability could be achieved in these cells by performing an additional PCI treatment **(**Fig. 2c**)**.

### synNF1 transduction reduces the amount of phosphorylated ERK1 in fibroblasts of NF1 patients

Neurofibromin plays a role in the mitogen activated protein kinase (MAPK) pathway as a GTPase activating protein increasing the conversion rate of Ras-GTP to Ras-GDP. Therefore, decreased amounts of neurofibromin were found to effect the phosphorylation of downstream effectors like ERK in the MAPK pathway^34,35^. Regarding this, we measured the amount of phosphorylated ERK1/2 in NF1 patient fibroblasts (NF244 and NF191, representing a good and a bad responder) before and after synNF1 transduction **(**Fig. 2d). Transduction without PCI treatment led to a small and not significant decrease in measured amounts of phosphorylated ERK 1 (pERK1) in trend. Though, a combination treatment of synNF1 transduction and PCI resulted in a high significant reduction of the measured amounts of pERK1. Levels of phosphorylated ERK2 did not change, neither using the single synNF1 transduction nor when performing a combination treatment. In NF1 wildtype control fibroblasts, synNF1 transfection either with or without additional PCI treatment did not change the levels of pERK 1 or 2 **(**Fig. 2e, left panel**)**.

## Discussion

Here, we describe the first successful transduction of the high molecular weight protein neurofibromin into the cytoplasm of living cells *in vitro*. Although several techniques have been invented to augment cellular uptake of synthetically produced proteins in the last few years, overcoming the endosomal entrapment and degradation still remains an essential issue. Fahrer *et al*.^38^ for example were able to transfer endosomally trapped proteins to the cytoplasm using a fusion complex consisting of core streptavidin and clostridial C2 toxin. Nonetheless, using this reagent shows a limitation to the proteins’ ability to being unfolded and refolded again correctly in the target cells^39^. An uptake of huge proteins as neurofibromin (~320 kDa) remains a challenging task. We showed that the problems can be overcome *in vitro* by using endo-lysosome disrupting techniques as specialized transduction reagents along with light triggered photochemical internalisation (PCI). As all of the methods may alter the proteins’ structure and function, it is important to verify if the functions are conserved during the treatments of the cells. To do this, we tested the impact of transduced neurofibromin on the MAPK pathway and the capability of the cells to orientate themselves to nano-micro structured surfaces. Both, a biochemical feature of neurofibromin and an effect on cellular behavior imply that the transduced neurofibromin or at least a significant amount of it retained the function of endogenous neurofibromin. The reduced orientation capability of *NF1*+/− fibroblasts to nano-micro structured surfaces was described few years ago^9^. These results were based on parallel measurements of fibroblasts either derived from healthy donors or NF1 patients. However, the degree of the so called partial blindness of *NF1*+/− fibroblasts varies between different patients. Therefore, we verified this novel effect of neurofibromin by performing siRNA based NF1 silencing in *NF1*+/+ fibroblasts. Transduction of the full-length neurofibromin rescued the phenotype of well orientating cells either in these NF1 silenced *NF1*+/+ fibroblasts and *NF1*+/− patient fibroblasts. One issue of the protein transduction is that the amount of uptaken proteins cannot be controlled precisely. Therefore it is not possible to transduce the exact amount of the missing endogeneous protein. This raises questions regarding potential toxicity or effective dosages and the applicability of protein transductions *in vivo*. *NF1* gene expression levels are generally low resulting in low levels of endogeneous neurofibromin. In concordance with our previous transduction experiments^31^, we used neurofibromin amounts comparable to the model drug Atto488-BSA. These amounts exceed the levels of endogeneous neurofibromin by far. Interestingly, we could not detect enhanced levels of dead cells or altered morphology of the cells by visual inspection, indicating a high tolerance to increased neurofibromin levels in fibroblasts. This may be due to a capping effect of the participation of neurofibromin. In the tested readout systems, neurofibromin transduction led to an effect limit that could not be overcome. The induced partial blindness by siRNA based *NF1* silencing could be rescued but not be outperformed. Just as well, the enhancement of the orientation capability in *NF1*+/− patient fibroblasts could be modulated by raising the release of the transduced neurofibromin using PCI. But an improvement of more than approximately 40% could not be reached, even when the initial transduction without PCI treatment already improved the orientation by 40%. This suggests a high resistance to high doses of neurofibromin in cultured fibroblasts and is supported by the missing effect on the amounts of phosphorylated ERK1 in synNF1 transduced and PCI treated control fibroblasts (Fig. 2e).

These proof of concept experiments give rise to possibilities to translate the transduction of neurofibromin to *in vivo* experiments with the goal of a restoring of lacking protein in all body cells. Here, additional issues have to be considered. Besides classical considerations concerning the bioavailability, form of application and camouflage techniques to prevent immune response more direct problems have to be solved. The applied transduction reagents and especially the PCI technique to enhance the endolysosomal escape are not suitable to be used *in vivo*. Whereas the bioavailability can be enhanced by PEGylation, the adaptation of PCI as a light induced treatment may be most complex. We used TPPS_4_ as photosensitizer (PS). This PS is activated by an exposure to visible blue light that penetrates tissue only over a distance of few millimeters with a strong decrease of the intensity. To perform a suitable PCI treatment, a uniform exposure in all cells should be achieved for that the effect of an endolysosomal release is directly linked to the light intensity. Here, the use of different PS with other activation wavelengths might extenuate this problem. Further modifications of the recombinant neurofibromin e.g. the fusion of one or more HIV TAT transduction domains might enhance the cellular and cytoplasmic uptake as well. This could obliterate the need of an external treatment and therefore pave the way for *in vivo* testing for the replacement of lacking very large proteins in monogenic diseases as such as NF1.

## Materials and Methods

### Cell culture

The processing of tissue and the preparation of the human fibroblasts was performed as described^32^. Biopsies from the healthy male donors (aged 3, 3 and 11 years, respectively) were obtained from the prepuce. Skin samples of NF1 patients (NF191 – NF329, aged 8–40 years) derived from NF1 patients clinically meeting the criteria for Neurofibromatosis type 1. The research carried out was in compliance with the Helsinki Declaration and approved by the local ethics committee (Ethikkommission University of Ulm, A 185/09). The patients gave written informed consent for scientific usage of their tissues. The fibroblasts were cultured in Dulbecco’s modified eagle medium (DMEM) with 10% fetal bovine serum (FBS), l-glutamin and antibiotics at 37 °C and 9% CO_2_.

### Expression and purification of synNF1 and synNF1eGFP

#### Cloning and generation of recombinant baculoviruses

A synthetic gene (GeneArt) encoding the human full-length NF1 (synNF1) was cloned into the pFastBacHta (Life Technology) vector in frame with the upstream His_6_-tag followed by a Tobacco Etch Virus (TEV-) protease cleavage site. For expression of full-length human neurofibromin with C-terminal eGFP tag (synNF1eGFP) the eGFP coding sequence was cloned three times subsequently in frame at the 3′-end of the full-length *NF1* sequence in the synNF1 pFastBacHta vector. Recombinant baculoviruses were generated according to Berger I. *et al*. using *E*. *coli* DH10Multibac cells containing the modified baculoviral genome and Sf21 insect cells (Life Technologies, Carlsbad, CA, USA).

#### Expression and purification

SynNF1 was expressed and purified as described^33,40^. For the production of synNF1eGFP Sf21 cells were infected with saturating amounts (multiplicity of infection (MOI) 1–2) of recombinant synNF1 or synNF1eGFP expressing baculoviruses, harvested via centrifugation after 72 h post-infection and stored at −80 °C. All subsequent steps were carried out at 4 °C. The cell pellet was resuspended in lysis buffer [50 mM HEPES pH 8.0, 300 mM NaCl, 10% glycerol, 5 mM TCEP, 10 mM imidazole] supplemented with cOmplete, EDTA-free protease inhibitor mix (Roche) and cells were lysed by a freeze thaw cycle in liquid nitrogen. Recombinant neurofibromin was purified from the soluble fraction via immobilized metal ion affinity chromatography (IMAC) using HisTrap FF columns (GE Healthcare) followed by a size exclusion chromatography (SEC) on a Superose 6 column (GE Healthcare) in 50 mM HEPES pH 8.0, 300 mM NaCl, 10% glycerol, 5 mM TCEP. SEC fractions containing synNF1 or synNF1eGFP were collected and concentrated using Vivaspin Centrifugal Concentrators with a cutoff of 100 kDa (Santorius). The purified protein was flash frozen in liquid nitrogen and stored at −80 °C.

### Transduction of synNF1 into fibroblasts

SynNF1 was transduced into fibroblasts using the transduction reagent EndoPorter (GeneTools, LLC). 5000 cells per cm^2^ were seeded in in 6-well plates. In the following, the fibroblasts were incubated in DMEM containing 10% FCS, L-glutamine, antibiotics, 18 µl Endoporter reagent and 9 µg of the synNF1 protein at 37 °C and 9% CO_2_ for 24 hours. According to the subsequent measurement, the cells were either trypsinized and seeded on PDMS gel (measurement of cell orientation), lysed in RIPA buffer (western blot) or examined using fluorescence microscopy (Zeiss, Axioscope).

### Western Blot

For western blot analyses, cells were lysed in RIPA buffer and 10–20 µg of total protein were used for SDS-PAGE. The proteins were transferred to PVDF membranes. For detection the following antibodies and the ECL Prime Western Blotting Detection Reagent (Amersham, GE Healthcare) were used: Anti- ERK1/2 (p44/42 MAPK (Erk1/2); Cell Signaling Technology; 1:1000), anti-phopho ERK1/2 (Phospho-p44/42 MAPK (Erk1/2) (Thr202/Tyr204); Cell Signaling Technology; 1:1000), anti-GAPDH; Santa Cruz Biotechnology Inc.; 1:3000), anti-rabbit IgG HRP (Santa Cruz Biotechnology Inc.; 1:25000) and anti-mouse IgG HRP (Santa Cruz Biotechnology Inc.; 1:50000). To compensate uneven gel loading, the detected protein bands were quantified using the ImageJ software (https://imagej.nih.gov/ij/). Before calculation of treatment effects corresponding bands were normalized using the measured amounts of the housekeeping protein GAPDH.

### Photochemical internalization (PCI)

The PCI treatment was performed as described previously^31^. In short, the cells were incubated for 24 hours in DMEM containing 1 µg/ml TPPS_4_ (5,10,15,20-Tetrakis-(4-sulfonato-phenyl)-21,23H-porphyrin, TriPorTech GmbH, Lübeck, Germany) to achieve an incorporation of the photosensitizer (TPPS_4_) into the fibroblasts’ membranes. To release subsequent transduced proteins, a 10 minute light exposure with visual blue light was performed (Osram L 18 W/67, Osram, München, Germany).

### siRNA transfection of human fibroblasts

The human *NF1*+/+ fibroblasts KF3 were transfected with 4 siRNAs (FlexiTube, Qiagen, Hilden; Supplementary Table 2) designed for silencing the NF1 message using the Lipofectamine RNAiMAX transfection reagent (Life Technologies). As a control the Allstar Negative Control siRNA (Qiagen) was used. The culture medium was removed and the cells were washed 2 times with phosphate buffered saline (PBS). The fibroblasts were trypsinized and centrifuged. Then, the cell pellet was resuspended in 2 ml DMEM and the cells were counted in a Neubauers’ chamber. The fibroblasts suspension was diluted resulting in a dilution of 50000 cells per 1.7 ml. For the production of the transfection complex 5 µl siRNA (10 µM; 1.25 of the 4 different NF1 siRNAs each or 5 µl of the control siRNA) were mixed with 292 ml OptiMEM (Gibco) and 3.3 µl Lipofectamine RNAiMAX followed by an incubation for 20 minutes at 20 °C. The 300.3 µl transfection complex was pipetted into a well of a 6-well plate (Nunc). Then 1.7 ml of the cell suspension (50000 cells) were mixed to the complex. After that, the fibroblasts were incubated with the transfection complex for 48 hours at 37 °C and 9% CO_2_.

### Relative quantification (RT qPCR) of the *NF1* transcripts

To quantify the *NF1* mRNA, total RNA was isolated either directly after the siRNA transfection was finished or after additional follow up times in DMEM at 37 °C and 9% CO_2_. For RNA isolation the RNeasy Mini kit (Qiagen) was used. The cells were transferred on ice, washed with PBS (4 °C) and then directly lysed in 700 µl of the kits’ RLT lysis containing 1% mercaptoethanol. The lysate was centrifuged through a shredder column (Qiagen) and then load on the kits’ RNA capture column. The following washing and elution steps were performed as indicated in the manufacturers’ handbook. Afterwards, 1 µg of the RNA was reverse transcribed into cDNA with the Superscript III kit (Qiagen) using random hexamers. In each qPCR reaction, 100ng RNA equivalent were used. Besides the *NF1* mRNA, *Alas1* and *PolR2A* mRNA levels were measured as housekeepers^41^. Each measurement was performed in triplicates. The relative amount of *NF1* mRNA was calculated using the geometric mean of both housekeepers to avoid gene specific fluctuations. The primer efficacies of the 3 primer pairs (primer sequences in Supplementary Table 2) were comparable allowing a relative quantification.

### Micro-nano-structured substrates

A master wafer was produced by conventional optical lithography. The desired lateral structure of the surface topography was transferred to a silicon wafer covered with photo resist by an optical illumination process. In the simplest case, one obtains the master wafer with the negative of the desired surface topography after appropriate developing steps. In the next step the surface topography was transferred to a polymer substrate. This was done by casting a polymere, poly(dimethyl)siloxane (PDMS) on the master wafer. The surface of the polymer substrate was now structured with the desired pattern. After further surface modification the polymer to make it adhesive for cells the substrate can be used to culture cells on it and to measure their behaviour. Polydimethylsiloxane (PDMS) was chosen as material for the cell culture substrates because of its biocompatibility. To fabricate microstructured PDMS substrates master wafers were produced by photolithography. Such master substrates with defined microstripe structures were used as the moulds for transferring the topography pattern to the PDMS substrates. In brief, the structured substrates were fabricated in a clean room as described previously^42^. Master substrates (2′ silicon-100 wafers) were structured by a photolithographic technique resulting in rectangular parallel grooves of a depth of 200 nm and a groove width and interspace of 2 µm. PDMS (Sylgard 184, Dow Corning) was polymerized by mixing the pre-polymer with the curing agent with a ratio of 10:1. The mixture was cured on the master wafer at 65 °C for 24 hours. Then, round experiment gels with a diameter of 0.7 cm were cut out of the master gel. Before cell experiments, PDMS substrates were sterilized with 100% ethanol, washed three times by PBS, transferred into 48well plates (Nunc), coated for 1 hour with FCS at 37 °C and 9% CO_2_ and finally used in the experiments. The surface topography of the PDMS substrates was verified by atomic force microscopy and scanning electron microscopy as previously shown^42^.

### Measuring the partial blindness of fibroblast to nanostructured PDMS gels

Cells were seeded on the PDMS substrates in regular culture media. After 48 hours, the cells were fixed in ethanol and methanol containing 2 µg/ml DAPI. After 2 washing steps in PBS, the PDMS substrates were examined on a Zeiss Axioscope. Images of the cells in phase contrast and the staining of the corresponding nuclei as fluorescence picture were taken. Microscopy images of the cells were analyzed by ImageJ software (http://rsb.info.nih.gov/ij/). The orientation angle of the cells was determined by using the orientation of the nucleus of the corresponding cell. The DAPI staining was used to highlight the nuclei and to fit ellipses. The angle between the principle axis of an ellipse and the nano-micro structures was used as the orientation angle of a single cell. At least 100 (up to 700) single cell orientation angles were measured. The median of the measured aberrations angles was used as the orientation of the cells in this experiment. For each treatment of the primary fibroblasts, 3–5 independent experiments were performed. Statistical analyses were performed by comparing the orientations of the independent experiment of differing treatment (e.g. NF1 status) using t-tests. The significance level was set to 0.05.

### Statistics

For statistical analysis, student’s t-tests were performed. A p-value < 0.05 was considered as significant.

## Electronic supplementary material

Supplementary Information
