# Supplementary material for: Restoring functional neurofibromin by protein transduction
Source: Sci Rep. 2018 Apr 18;8:6171. doi: 10.1038/s41598-018-24310-5 (PMC5906691; doi:10.1038/s41598-018-24310-5)
Supplement: Supplementary file 1 — Supplementary Information [file 41598_2018_24310_MOESM1_ESM.pdf]

Supplementary Information

Restoring functional neurofibromin by protein transduction

K. Mellert[1*,3†], S. Lechner[2], M. Lüdeke[3], M. Lamla[4], P. Möller[1], R. Kemkemer[5,6], K. Scheffzek[2†], D. Kaufmann[3,6*†]

1 Institute of Pathology, University of Ulm, Ulm, Germany

2 Division of Biological Chemistry, Biocenter, Innsbruck Medical University, Innsbruck, Austria

3 Institute of Human Genetics, University of Ulm, Ulm, Germany

4 Institute of Organic Chemistry III, Ulm University, Ulm, Germany

5 Department of New Materials and Biosystems, Max Planck Institute for Medical Research, Stuttgart, Germany

6 Reutlingen University, Applied Chemistry, Reutlingen, Germany

† Corresponding author: Kevin.mellert@uni-ulm.de, Klaus.scheffzek@i-med.ac.at, Dieter.h.kaufmann@uni-ulm.de

* present affiliation

Supplementary Figure 1

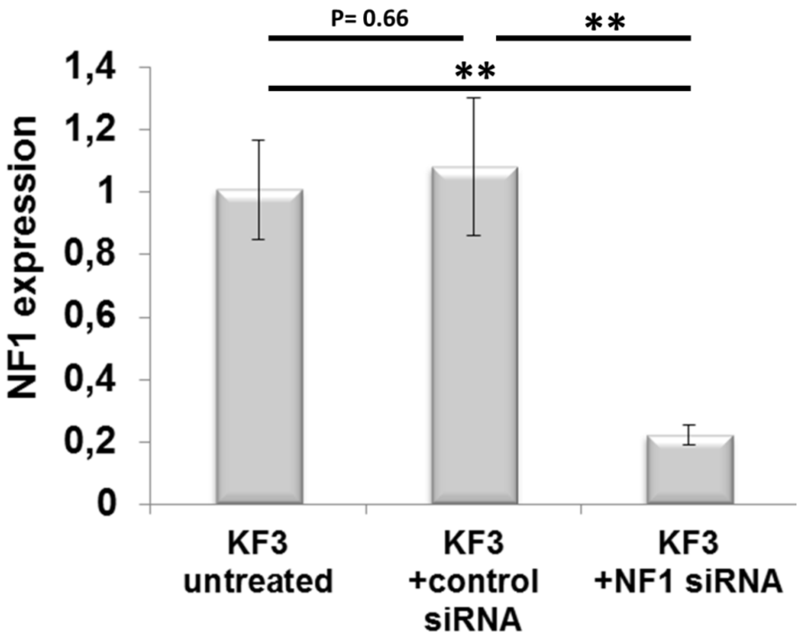

Supplementary Figure 1: NF1 knockdown by siRNA transfection. Shown are the levels of measured amounts of NF1 mRNA in KF3 fibroblasts either untreated or after 48 hours of transfection with control siRNA (AllStars Negative Control siRNA, Qiagen, Hilden; KF3 + control siRNA) or 4 NF1 specific siRNAs (KF3 +NF1 siRNA; means of 3 independent experiments). The error bars represent +/- 1 standard deviation. Stars indicate the level of significance (** = p<0,01; two-sided T-test).

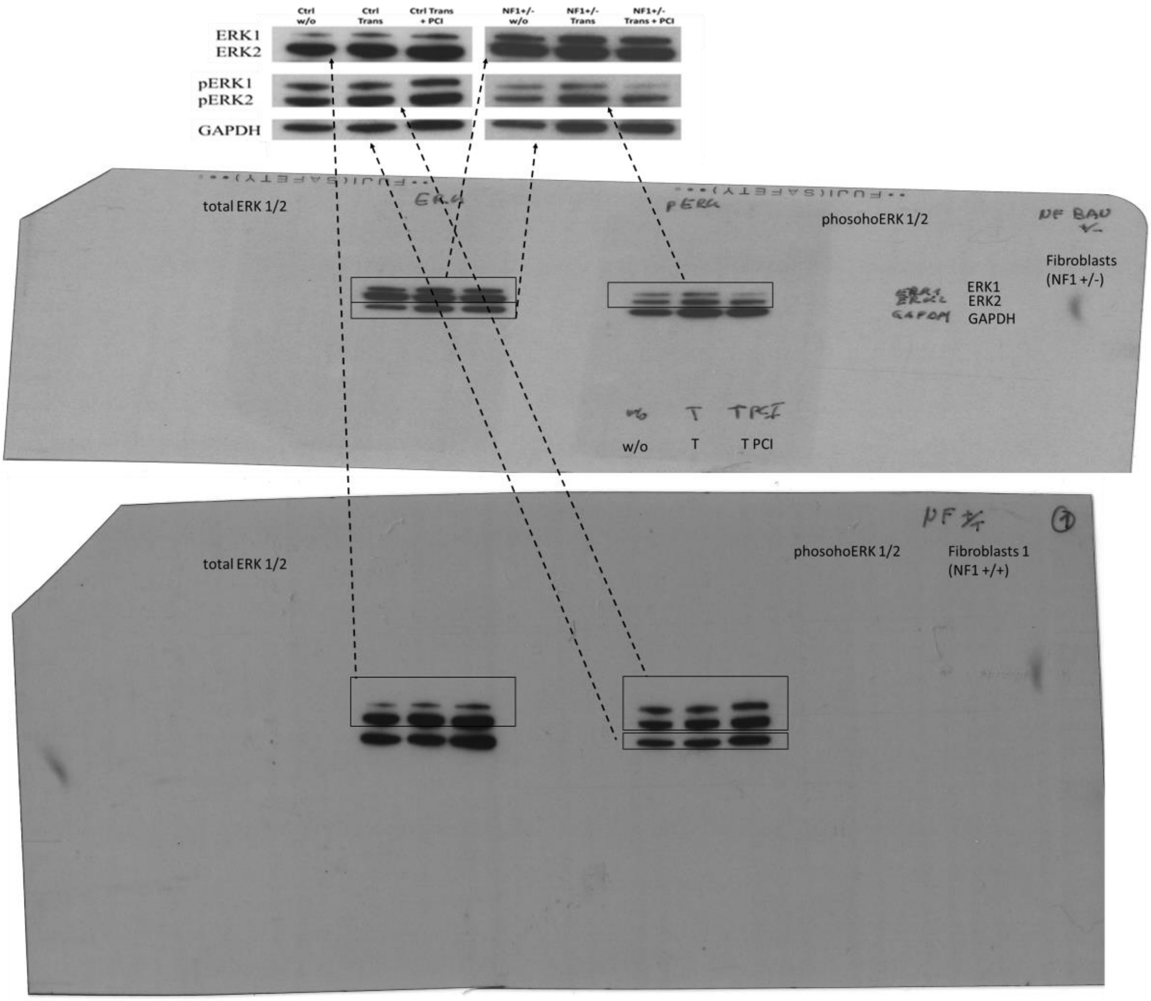

Supplementary Figure 2: Original western blot detections. The bands were cut and rearranged from these two photo films as indicated by rectangles and arrows. Annotations were added to enhance the readability of handwritten notes on the original films.

Supplementary table 1: Primer and siRNA sequences. Shown are the primer/siRNA names, target genes, sequences, annealing temperatures (AT) and product lengths (P).

Name	***Gene***	***Sequence (5‘-3‘) / siRNA ID***	***AT***	***P***
NF1 fwd	NF1	GCAGTTCTGACCCGAGTTTACG	60°C	99 bp
NF1 rev	NF1	ATGTCTCTAGTAACTGGCCCTCGAT	60°C	
POLR2A fwd	POLR2A	GTTTTGGTGACGACTTGAACTG	59,5°C	145 bp
POLR2A rev	POLR2A	CGCAGGAAGACATCATCATC	59,5°C	
ALAS1 fwd	ALAS1	TGATGAACTACTTCCTTGAGAATC	60°C	70 bp
ALAS1 rev	ALAS1	GAATGAGGCTTCAGTTCCA	60°C	
siNF1 1	NF1	SI02664445 (FlexiTube, Qiagen)	-	-
siNF1 2	NF1	SI02664438 (FlexiTube, Qiagen)	-	-
siNF1 3	NF1	SI04949938 (FlexiTube, Qiagen)	-	-
siNF1 4	NF1	SI04949931(FlexiTube, Qiagen)	-	-
